# Language-related acculturative hassles and their association with mental health of minors with refugee background living in Germany

**DOI:** 10.3389/fpsyg.2025.1673185

**Published:** 2025-11-06

**Authors:** Johanna Braig, Pia Schmees, Yasemin Kilinc, Usama EL-Awad, Hannah Nilles, Denny Kerkhoff, Jana-Elisa Rueth, Arnold Lohaus, Heike Eschenbeck

**Affiliations:** 1 Department of Educational Psychology and Health Psychology, University of Education Schwäbisch Gmünd, Schwäbisch Gmünd, Germany; 2 Department of Psychology, Bielefeld University, Bielefeld, Germany; 3 Department of Clinical Psychology and Psychotherapy, Medical School Berlin, Berlin, Germany; 4 Department of Psychology, University of Münster, Münster, Germany

**Keywords:** language hassles, mental health, refugee children and adolescents, acculturation, language proficiency

## Abstract

**Introduction:**

The public debate on migration often focuses on refugees’ cultural adaptation, with language barriers being a key concern. Previous research demonstrated associations of mental health with acculturative hassles but there is little longitudinal research. Therefore, this study examines the longitudinal relationship between the mental health of refugee minors and language-related acculturative hassles concerning the host country’s language. We assume that internalizing symptoms are associated with language hassles and predict them over time, for externalizing symptoms no assumptions were made.

**Methods:**

A non-clinical sample of 11–19-year-old minors with refugee background living accompanied in Germany (*n* = 63) answered self-report questionnaires on mental health, language proficiency, and language related hassles twice at intervals of approximately 1 year.

**Results:**

Baseline language hassles and internalizing symptoms (Δ*R*^2^ = 0.06) but not externalizing symptoms predicted follow-up language hassles.

**Discussion:**

Our study shows initial, longitudinal indications that impaired mental health may be a risk factor for language hassles. This is considered important for affected minors and their caregivers, e.g., for education and peer contact. Replication of the findings with a larger sample size is recommended.

## Introduction

Around 123 million people were forcibly displaced in 2024. With 2.7 million refugees living in Germany, it represents one of the largest host countries for people who have fled across national borders (United Nations High Commissioner for Refugees, 2025). Refugee minors (RM) are a vulnerable group: They are likely to have experienced potentially traumatic life events before or during their escape and post-migration stressors after their arrival in the host countries, both associated with symptoms of impaired mental health (Dangmann et al., 2021; EL-Awad et al., 2021; Müller et al., 2019; Scharpf et al., 2021). Acculturative hassles are a subtype of post-migration stressors (Sirin et al., 2013; Titzmann et al., 2011; Vinokurov et al., 2002). Following the established distinction between daily hassles and major life events, acculturative hassles are conceptualized as minor, chronic or repeated strains that arise from experiences related to the acculturation process (e.g., concerning ethnic discrimination, language of the host country or family, DeLongis et al., 1982; Titzmann et al., 2011; Vinokurov et al., 2002). As for other post-migration stressors, associations of acculturative hassles with mental distress were reported (Keles et al., 2016; Keles et al., 2017; Lincoln et al., 2016; Vinokurov et al., 2002). Language hassles describe subjectively perceived challenges related to the language of the host country, with a focus on the social context (e.g., school). They can result from insufficient competence in the host country’s language but are assumed to additionally depend on the intensity of contact with the host culture (Titzmann et al., 2011). Even a proficient speaker might subjectively perceive hassles in certain situations (e.g., “I was ashamed of my German, so I did not date a local boy/girl”, Titzmann et al., 2011). Language hassles are assumed to be particularly relevant for minors, given that the language of the host country forms the basis of the education system.

The concept of language proficiency is overlapping but distinct to language hassles; Language proficiency is often seen as a complex construct involving a range of linguistic competencies and skills (e.g., communicative skills and competencies as vocabulary or grammar; Harsch, 2017). Various frameworks as the Common European Framework of Reference for Languages (in particular for adults, Council of Europe, Council for Cultural Co operation, Education Committee, Modern Languages Division, 2001) attempt to conceptualize objectifiable levels of proficiency.

### Why do some RM struggle more with the language of the host country than others?

Language competencies are seen as crucial for interpersonal (e.g., social communication, education) and internal (e.g., self-regulation) adaptation processes and thus the development of both, monolingual and bilingual children (for an overview, see Toppelberg and Collins, 2010). While most children quickly acquire the language of their host country, some struggle (see for example Gambaro et al., 2025; data on children who came to Germany from Ukraine). It is therefore essential to understand why some RM experience more challenges when learning a new language and report more language hassles than others. A frequently referenced model of language acquisition describes second language acquisition as a function of exposure to the host country’s language, learning efficiency and economic incentives (Chiswick and Miller, 2001), whereby for children non-economic incentives (e.g., benefits related to education) might be more relevant (Seuring and Will, 2022). Individual characteristics such as duration of stay in the host country can be embedded in this model (Chiswick and Miller, 2008; van Tubergen, 2010). Several studies investigated specific personal and contextual factors for refugee populations in second language acquisition, with mental health discussed as one aspect among others (Bernhard and Bernhard, 2021; Kosyakova et al., 2021; Plutzar, 2016; Seuring and Will, 2022; van Tubergen, 2010).

### Mental health and language outcomes

Since few studies have examined the relationship between mental health and language hassles, we first summarize findings on the relationship between mental health and other language outcomes (e.g., proficiency). Numerous correlational and a few longitudinal studies reported associations between mental health outcomes and language proficiency, predominantly in adult migrant populations (see Montemitro et al., 2021).

For children in general, there is meta-analytic data indicating small effect sizes for the association of internalizing as well as externalizing behavior with language difficulties (for an overview, see Hentges et al., 2021). But few studies with RM populations investigated the links between mental health and language outcomes. A review including only RM found four studies on adaptation processes and language proficiency; two showed an association, two showed no significant results (Scharpf et al., 2021). For RM in Germany, negative correlations between depressive symptoms as well as symptoms of PTSD and language proficiency were reported (Meyer et al., 2023; Müller et al., 2019). However, in the study by Müller et al. (2019), the correlations with the superordinate concepts of externalizing and internalizing symptoms were not significant. Moreover, another study did not find a significant association between the parents reported risk of PTSD and language competencies (Seuring and Will, 2022).

### Mental health and language hassles

So far only few studies explored the postulated relationship between mental health and language hassles: In a cross-sectional study with Soviet RM living in the US Vinokurov et al. (2002), found an association between mental distress (symptoms of depression and anxiety) and language hassles Nair et al. (2013) found an association between baseline language hassles and increased internalizing and externalizing symptoms at a 2-year follow-up for Latino minors living in the US. Only internalizing but not externalizing symptoms were associated with language hassles at baseline (Nair et al., 2013). A study with adolescents of immigrant descent living in Germany found an association between psychological distress (physiological stress and depressive symptoms) and language hassles (Kunyu et al., 2021).

### Underlying mechanisms for the association between mental health and language outcomes

Bidirectional mechanisms are plausible (language outcomes may impact mental health and vice versa). Given the high average mental burden of RM, our focus is on mechanisms that could explain pathways through which impaired mental health as a risk factor for RM may contribute to language hassles. So far, possible underlying cognitive and affective mechanisms are poorly researched but theoretically inferable: Firstly, RM show an elevated prevalence of mental disorders such as depression, anxiety and PTSD compared to the general population (Blackmore et al., 2020; Kien et al., 2019). Some symptoms of these disorders are known to affect cognitive processes that are relevant for language learning in different ways, for example due to impaired motivation or reduced concentration (Iversen et al., 2014; Kaplan et al., 2016).

With regard to internalizing disorders, which are (by definition) accompanied by altered affective processes such as feelings of worthlessness and negative self-image (American Psychiatric Association, 2013; World Health Organization, 2019), it can be assumed that the social dimension of language acquisition is particularly relevant. According to socio-cognitive models, second language learning is taking place in social interactions (Atkinson, 2004; Li and Jeong, 2020). Consequently, problems in interpersonal situations and emotions such as anxiety are relevant for language learning but were less researched than cognitive aspects (Dewaele and Li, 2020; Swain, 2013). It might be hypothesized that impaired social cognition could be an underlying mechanism for both, mental disorders and impaired language learning (Montemitro et al., 2021). Thus, internalizing symptoms can be considered a plausible risk factor for impaired language outcomes at all levels of Chiswick and Miller’s (2001) model of second language acquisition: For example, reduced exposure due to social withdrawal, reduced effectiveness due to concentration deficits, reduced incentives due to loss of interest in social contacts.

For externalizing symptoms and their relationship with language outcomes it is difficult to draw conclusions based on the existing literature with non-refugee populations. Explanations often focus on language difficulties or language disorders as risk factor for externalizing symptoms (Petersen and LeBeau, 2021; Toppelberg and Shapiro, 2000). However, a generalization of this reasoning to RM with difficulties in the host country’s language but unproblematic first language development may be inappropriate: When the first language is unimpaired, it should be available for self-regulation and family communication, aspects discussed as important pathways for the reported association between language outcomes and mental health in general population children (Toppelberg and Collins, 2010).

### The present study

This study contributes to the understanding of factors associated with the so far insufficiently investigated construct of language hassles in RM. The study assumes that language hassles and language proficiency are negatively correlating, overlapping but distinct constructs that relate to mental health outcomes in different ways. The main objective of the study is to examine whether certain mental health symptoms at baseline are associated with language hassles at follow-up. Given the evidence of previous studies subsumed above, we hypothesize that higher levels of internalizing symptoms (depression and anxiety) at baseline predict a higher score of language hassles at follow-up, when relevant covariates are considered. For externalizing symptoms, one study highlighted language hassles as predictor for externalizing symptoms (Nair et al., 2013). But since this approach is vice versa to the pathway we investigate in our study, the existing evidence is not clearly indicating an effect of externalizing symptoms on language hassles at follow-up. Consequently, this study explorative examines the role of baseline externalizing symptoms for follow-up language hassles.

For the regression analyses, language hassles and language proficiency (due to conceptual overlap) at baseline were considered as potential covariates. In addition, we considered demographic variables that had been associated with the predictors (mental health) or with language outcomes in previous studies: age (Wong and Edwards, 2023; Kieling et al., 2024), gender (Bernhard and Bernhard, 2021; Hentges et al., 2021; Kieling et al., 2024), and duration of stay in the host country (Bernhard and Bernhard, 2021; Kosyakova et al., 2021).

## Method

### Sample

The data for this study was collected in three waves in annual intervals between 2019 and 2022 within the YOURHEALTH collaborative project funded by the German Federal Ministry of Research, Technology and Space. Participants were recruited in their accommodations, schools, sports- and youth clubs in three regions in Germany. Table 1 provides an overview of the demographic characteristics of the non-clinical sample, living accompanied in Germany (*n* = 63).

**Table 1:** Socio-demographic characteristics of participants (*n* = 63).

Age (in years)^a^, *M (SD)*	14.87 (1.90)
Gender, *n* (%)	
Male	32 (51)
Female	31 (49)
Duration of stay in Germany (estimated in years)^a^, *M (SD)*	2.52 (1.27)
Country of origin, *n* (%)	
Syria	42 (67)
Iraq	13 (21)
Afghanistan	8 (13)

a Baseline assessment.

### Procedure

Written informed consent was obtained from parents or guardians and participants before the start of the study. Participants received a voucher for each participation. The study was approved by the ethics board of the participating universities (University of Bielefeld, 2018-161, University of Education Schwäbisch Gmünd, 2018-03). The questionnaire was available either in paper-pencil or as a digital questionnaire (QuestionPro, 2019). Participants could choose the language of the questionnaire (German, Arabic, Dari, Kurmancî, Sorani, or Pashto) and had the option to listen to the questions to ensure data quality even with low literacy skills. To guarantee appropriate support for the participants, the initial data collection took place in person (in a group setting), while follow-up appointments (approximately 1 year later) were also supervised by telephone. Upcoming questions or burden during or after data collections could be discussed with trained staff, preferably in the participants’ native language (either in person or via phone). Moreover, participants received information on whom to contact for mental health support.

### Measures

All questionnaires were self-report measures. Validated translations of the Hopkins Symptom Checklist-37A for Adolescents (HSCL-37A) were used as provided by the authors (Bean et al., 2004). All other questionnaires used in this study were translated and back-translated by native speakers and/or professional translators and checked for inconsistencies in collaboration with psychologists.

#### Language hassles (baseline and follow-up)

Language hassles were assessed using the language subscale of the Acculturative Hassles Questionnaire (Titzmann et al., 2011). The questionnaire measures different types of acculturative hassles, whereby the language hassles subscale comprises six items (e.g., *problems in class because my German was not good enough*). Participants rated on a 5-point response scale from *never* to *more than 10 times in the last 12 months*, how often they experienced each hassle (Cronbach’s *α* = 0.88).

#### Language proficiency (baseline)

German language proficiency was measured with four items asking for the ability to speak, understand, read, and write German (*α* = 0.91). Participants could answer on a 4-point response format from *not at all* to *very good*.

#### Internalizing symptoms (baseline)

Symptoms of depression and anxiety were assessed using the internalizing subscale of the HSCL-37A (Bean et al., 2007). For each of the 25 items, the participants rated on a 4-point scale from *never* to *always*, how often they had felt as described in the last month (*α* = 0.92).

#### Externalizing symptoms (baseline)

Externalizing symptoms (i.e., symptoms of conduct disorder and of oppositional-defiant disorder) were assessed using the externalizing subscale of the HSCL-37A (Bean et al., 2007). For each of the 12 items, the participants rated on a 4-point scale from *never* to *always*, how often they have felt as described in the last month (*α* = 0.63).

### Statistical analyses

Data were analyzed using IBM SPSS Statistics 29. Participants were eligible for the subsequent analyses if they stated that they had arrived in Germany during or after 2015, reported Syria, Iraq, or Afghanistan as country of origin, were between 11 and 19 years old at primary assessment and received the complete questionnaire at least twice (during the COVID pandemic also shortened versions of the questionnaire were used). This results in a total of *n* = 95 possible participants.

For the HSCL-37A, missing values (externalizing symptoms up to one, internalizing symptoms up to two missing item values), were replaced by extrapolation of the mean value as recommended by the manual (see Bean et al., 2004). No further imputation strategies were applied. After replacement of missing values, *n* = 27 participants were excluded because of missing data on the primary outcome variable language hassles and *n* = 5 because of missing data on one of the predictor variables. This results in *n* = 63 participants. As part of the preliminary analyses, participants with and without complete data for the primary outcome (follow-up language hassles) were compared using a *χ*^2^-test and independent *t*-tests. There were no significant differences between participants with completed language hassles questionnaire data and those excluded because of missing values regarding age, gender or duration of stay in Germany. Bivariate correlations revealed no significant correlations for age, gender, and language proficiency with the primary outcome (Table 2). Consequently, to simplify the model and accommodate the rather small sample size, these variables were not included as control variables. A power analysis was conducted using G*Power 3.1 (Faul et al., 2009). A required sample size of *n* = 68 for a medium effect of *f*^2^ = 0.15 was calculated for the remaining four predictors and *R*^2^ increase of two predictors (internalizing and externalizing symptoms), testing with an *α* error probability of <0.05 and a power of 1 − *β* error probability of 0.80. Relevant assumptions for the regression model were checked and no assumptions were markedly violated.

**Table 2:** Correlations of study variables.

Variable	1	2	3	4	5	6	7
1. Language hassles^a^^,^^c^ (follow-up)	—						
2. Language hassles^a^^,^^c^ (baseline)	0.51^**^	—					
3. Language proficiency^a^^,^^c^ (baseline)	−0.10	−0.33^**^	—				
4. Age (baseline)^a^^,^^c^	−0.02	0.13	−0.09	—			
5. Gender^a^^,^^b^^,^^d^ (baseline)	0.01	−0.03	−0.16	−0.10	—		
6. Duration of stay in Germany^a^^,^^c^ (baseline)	−0.28^*^	−0.40^**^	0.51^*^	−0.10	0.21	—	
7. Internalizing symptoms^a^^,^^c^ (baseline)	0.41^**^	0.38^**^	0.08	0.24	−0.24	−0.14	—
8. Externalizing symptoms^a^^,^^c^ (baseline)	0.21	0.11	0.17	0.10	−0.16	−0.05	0.69^**^

a *n* = 63.

b 0 = female, 1 = male.

c Pearson correlation coefficient.

d Point-biserial correlation coefficient.

^*^p < 0.05, ^**^p < 0.01 (2-tailed).

For the main analyses, we conducted a step-wise hierarchical linear regression analysis with language hassles at follow-up as outcome and baseline levels of internalizing and externalizing symptoms as predictor. Control variables were entered in step 1 (duration of stay in Germany) and step 2 (baseline language hassles). In order to account for the small sample size and given the high correlation of the predictors, the predictor variables were entered stepwise in step 3 if they had a *p*-value ≤ 0.05.

## Results

Descriptive statistics for the symptom scales and for language hassles are reported in Table 3. On average, participants reported having experienced the respective language hassles once or twice in the past year, with *problems at school* and *not being able to explain what they want to say* being experienced most frequently. The bivariate correlations for all variables are given in Table 2. As expected, baseline language proficiency and baseline language hassles were negatively correlated.

**Table 3:** Descriptive statistics for mental health and language hassles.

Internalizing symptoms (range: 25–100)^a^, *M (SD)*	39.05 (10.72)
High level of internalizing symptoms (score ≥ 54)^a^^,^^b^, *n* (%)	7 (11)
Externalizing symptoms (range: 12–48)^a^, *M* (*SD*)	14.52 (2.45)
High level of externalizing symptoms (score ≥ 19)^a^^,^^b^, *n* (%)	4 (6)
Language hassles (range: 6–30)^a^, *M (SD)*	12.63 (6.44)

a Baseline assessment.

b As described in other studies, we used percentile scores (60th and 90th percentile for internalizing and externalizing symptoms respectively) derived from data of unaccompanied refugee minors as indicators for high burden (Bean et al., 2007; Jensen et al., 2015; Müller et al., 2019).

### Baseline internalizing and externalizing symptoms as predictor for follow-up language hassles

The results of the regression analysis are reported in Table 4. Adding internalizing symptoms as predictor resulted in a 6% increase in explained variance. In the final model, baseline language hassles [*β* = 0.37, 95% CI (0.11, 0.58), *p* = 0.004] and baseline internalizing symptoms [*β* = 0.26, 95% CI (0.01, 0.27), *p* = 0.031] predicted a higher level of language hassles in RM at the follow-up after about 1 year, *R*^2^ = 0.32, *F*(3, 59) = 9.41, *p* < 0.001. The hypothesis, assuming that the baseline level of internalizing symptoms predicts follow-up level of language hassles was confirmed. The baseline level of externalizing symptoms did not predict the follow-up level of language hassles.

**Table 4:** Hierarchical linear regression analysis for language hassles at follow-up as outcome and baseline internalizing and externalizing symptoms as predictor.

Variable	*B*	95% CI for *B*		*SE B*	*β*	*R* ^2^	Δ*R*^2^
		*LL*	*UL*				
Step 1 (enter)						0.08	0.08^*^
Constant	15.03^***^	11.77	18.29	1.63			
Duration of stay in Germany (baseline)	−1.34^*^	−2.50	−0.18	0.58	−0.28^*^		
Step 2 (enter)						0.27	0.19^***^
Constant	7.31^**^	2.39	12.23	2.46			
Duration of stay in Germany (baseline)	−0.46	−1.60	0.67	0.57	−0.10		
Language hassles (baseline)	0.44^***^	0.21	0.66	0.11	0.47^***^		
Step 3 (stepwise)						0.32	0.06^*^
Constant	2.94	−3.25	9.14	3.10			
Duration of stay in Germany (baseline)	−0.48	−1.58	0.62	0.55	−0.10		
Language hassles (baseline)	0.35^**^	0.11	0.58	0.12	0.37^**^		
Internalizing symptoms (baseline)	0.14^*^	0.01	0.27	0.06	0.26^*^		
Externalizing symptoms (baseline)	Excluded						

CI, confidence interval; LL, lower limit; UL, upper limit. Excluded in step 3 (*p*-value > 0.05): externalizing symptoms (baseline).

^*^p < 0.05, ^**^p < 0.01, ^***^p < 0.001.

## Discussion

This study adds evidence to the existing literature by focusing on mental health symptoms and their association with follow-up language hassles in a sample of RM, for whom there is little data in this context to date. In the final model of a hierarchical multiple regression, baseline language hassles and internalizing symptoms but not externalizing symptoms predicted follow-up language hassles. Thus, in our sample, RM with more internalizing mental health difficulties were not only burdened regarding the specific symptoms of their mental health problems but were also at risk to face long-term challenges regarding language. This finding is in line with various correlational studies showing an association between symptoms of depression and anxiety with language outcomes (Montemitro et al., 2021).

This finding is of practical relevance at various levels. Firstly, language difficulties were perceived as a major source of stress in a qualitative study with RM, affecting education and social contacts (Poppitt and Frey, 2007). Thus, early intervention might prevent a vicious circle of impaired mental health and language difficulties. Secondly, language hassles could be a reason why RM struggle at school. Again, adequate early intervention for minors at risk might prevent further educational disadvantages. This is of relevance, as in the German education system RM often attend lower school types than the general student populations (Will and Homuth, 2020).

We found no effect of externalizing symptoms on language hassles, despite the high correlation of internalizing and externalizing symptoms. As described in the introduction, some mechanisms discussed as potentially relevant mediators for the relationship between mental health and language outcomes in general populations refer to processes that are specifically relevant to the first language. For example, the importance of language in self-regulation may not be applicable for the situation of RM, since their first language may be available for this purpose. Moreover, explanations often focus on the reverse pathway (i.e., language difficulties as risk factor; Toppelberg and Shapiro, 2000). For other mediating mechanisms discussed above, such as motivation and social withdrawal, it is conceptually plausible in terms of diagnostic criteria that they are more closely related to incremental variance components of internalizing symptoms than to a general impairment of mental health. The stepwise analysis strategy is not the explanation for the lack of effect of externalizing symptoms, since two individual regression analyses produced comparable results.

Baseline language proficiency and baseline language hassles were negatively correlated. Nevertheless, there was no significant association between language proficiency and mental health outcomes. This finding is consistent with previous studies that found no significant association between language outcomes and mental health measures (Kosyakova et al., 2021; Seuring and Will, 2022). However, it contrasts with the majority of studies in refugee and migrant populations, which report associations between language and mental health outcomes (e.g., Meyer et al., 2023; Montemitro et al., 2021; Müller et al., 2019). The relatively low percentage of highly distressed participants in our sample may partly explain the deviating results, as effects related to mental health might be more difficult to detect in a less burdened population. The plausibility of our finding is further supported by the conceptual distinction between the two language related constructs. The language hassles scale reflects more social aspects of language (e.g., *I felt alienated in Germany, because my language abilities are not sufficient*). These social aspects may be particularly impaired in minors with depressive symptoms, for example due to impaired self-efficacy (Bandura et al., 1999). In line with the assumption that social aspects are relevant for the association between internalizing symptoms and language hassles Kunyu et al. (2021), found that the relationship between language hassles and psychological distress was mediated through belonging with classmates.

Moreover, the hassles scale has a strong focus on individual perception. Consequently, a greater susceptibility of the language hassles scale to a depression-related negative view of oneself and of one’s environment (e.g., higher sensitivity to hassles even with comparable proficiency level) in terms of Beck’s cognitive triad is plausible (Beck et al., 2024). Further studies could examine whether the postulated association of impaired mental health with language hassles is mediated by the discussed mechanisms.

Another explanation for the non-significant associations between language proficiency and mental health could be that a relevant proportion of our sample lived in refugee accommodations. The home language environment there may have attenuated a potential effect of reduced host country’s language skills (discussed in a similar way by Gormez et al., 2018). The hassles scale could have more specifically captured the challenges that nevertheless exist.

### Strengths and limitations

Given the limited sample size, the power might not have been sufficient to detect small effects (e.g., a possible effect of externalizing symptoms) and possible overfitting of the model cannot be completely ruled out. Moreover, there was not enough data available on PTSD symptoms to include PTSD as predictor variable.

The externalizing subscale showed a rather low reliability, which limits the conclusiveness of the results. In earlier studies and also in a study with a larger, partially overlapping sample (EL-Awad et al., 2021), the scale has proven to be reliable, so that the reliability of the scale is not fundamentally in question.

Another limitation arises from the fact that, due to the study design, our language proficiency scale was a self-report scale, which significantly limits its objectivity. Future studies with larger samples could apply cross-lagged panel models to more thoroughly examine the probable directionality of effects. The question, if impaired mental health is also associated with other types of acculturative hassles is beyond the scope of this article, but interestingly Vinokurov et al. (2002) found that the correlation between language hassles and mental distress was the highest correlation for all acculturative hassles domains assessed, which emphasizes their importance. In order to avoid our model becoming too complex for the small sample, we did not include language proficiency in the first language, although it can be assumed that the balance of both languages is also relevant for adaptation processes (Vedder and Virta, 2005). Furthermore, we cannot determine the sequence in which the participants acquired their languages, or the function of the host country’s language. Therefore, we refer to a “second language” as any language acquired after the first language has been learnt in the early years of childhood (Mitchell et al., 2019). When interpreting the results, it is important to emphasize that we only surveyed a small subset of the diverse group of RM, namely a non-clinical sample of 11–19-year-olds from Syria, Iraq and Afghanistan who are currently living in Germany. It would not be appropriate to generalize the results to other samples especially to clinical or potentially more distressed samples such as unaccompanied RM.

However, there are various strengths of this study. In view of the insufficient longitudinal data available to date and the challenges associated with the sampling process (frequent relocations, unsecure residential status) the longitudinal design represents a major strength. In the context of a one-year longitudinal study with an assumed complex set of influencing variables, the effect found for Δ*R*^2^ of 0.06 can be classified as small, yet presumably of practical relevance. Although language difficulties are a frequently discussed topic in the context of migration, there is no established scale for measuring them. One of the strengths of our study is the parallel recording of language hassles and proficiency, which provides a broader insight into both concepts and the perceptions of the minors concerned. A further strength is the differentiated assessment of mental health with commonly used measures for RM. In addition, our sample was collected in various settings in different regions of Germany, so that a high external validity can be assumed.

## Conclusion

To date, there is little data on the relationship between language outcomes and mental health in RM, although both topics are relevant issues in the public debate on migration with potential relevance for the educational pathway. Our research is contributing to the field by showing a significant longitudinal association of both concepts. More specifically, language hassles at baseline and internalizing but not externalizing symptoms were predictive for language hassles at follow-up. Considering the results of our study as well as previous studies (e.g., Montemitro et al., 2021; Nair et al., 2013), bidirectional relationships between mental health and language outcomes seem plausible. Due to the limited sample size and power, as well as the study design, causal conclusions cannot be drawn, and small effects (e.g., a possible effect of externalizing symptoms) may remain undetected. Therefore, larger studies should investigate the effects of specific language-related and psychotherapeutic interventions on language hassles and mental health in minors. The findings are relevant to practitioners in the fields of education and health since they provide an initial indication that it can be valuable to consider whether reported language hassles may be related to impaired mental health. In this way, affected children can be referred to support services if necessary.

## Data Availability

The raw data supporting the conclusions of this article will be made available by the authors without undue reservation.
